# Tiny lives, big failures: India's drug regulation crisis

**DOI:** 10.1016/j.lansea.2025.100705

**Published:** 2025-12-19

**Authors:** The Lancet Regional Health – Southeast Asia

In October 2025, WHO issued a product alert for three substandard (contaminated) oral liquid medicines manufactured and marketed in India resulting in the death of 20 children. The incident involved contamination of the excipients propylene glycol and glycerine, which are commonly used in cough formulations, with the toxic industrial chemicals diethylene glycol (DEG) and ethylene glycol (EG). Since 1937, over 1000 deaths have been reported globally due to contaminated medicines. The first major incident involving DEG/EG prompted the creation of the US Food and Drug Administration (FDA) and its stringent drug safety standards. While regulations have tightened in high-income countries, these events continue to harm lives in low-income and middle-income countries. Since 2022, WHO has issued seven alerts concerning contaminated liquid oral medicines, almost all of which had manufacturing located in India and the majority of which reported serious adverse events, including over 300 deaths.

These concerns warrant a closer look into the country's regulatory landscape. The key regulatory authority for the country is the Central Drugs Standard Control Organisation (CDSCO), under the Ministry of Health, headed by the Drug Control General of India (DCGI). CDSCO operates in coordination with state and district drug control offices and is supported by drug inspectors at the ground level. It maintains a network of drug testing laboratories to ensure quality standards. Its operations are also governed by the Drugs and Cosmetics Act (DCA), which provides legislation required for pharmaceutical regulation. In response to several quality concerns, the DCA was recently amended and schedule M was also revised with stringent penalties, mandatory inspections, and a requirement for provision of proof of stability and safety of excipients by the manufacturers in line with WHO Good Manufacturing Practices (GMP). But implementation remains inadequate, as indicated by the government's continuing extension of the timeline for local manufacturers to comply with this legislation. It is also commendable that imprisonment terms and penalties have been tightened in the new Drugs, Medical Devices and Cosmetics Bill, 2022.

Although the country has brought in many legal changes to address gaps, without robust enforcement, ensuring legal compliance remains unattainable. A 2012 parliamentary report which highlighted CDSCO's deficiencies prompted a *Lancet* editorial to even call for its complete overhaul. Over a decade later, concerns about CDSCO prioritising economic goals over patient safety remain and are now more alarming than ever. The 2022 tragedy in The Gambia, where 66 children died from contaminated imported Indian cough syrups, was followed by the introduction of quality control procedures for exported drugs. These included mandatory pre-export testing and a requirement from the Ministry of Commerce for exporters to submit certificates of analysis. However, domestic drug manufacturing continues to face weak regulatory mandates.

The existing mechanism suffers from major shortcomings in both structure and implementation. While organisations like the FDA and European Medicines Agency are well established with tight regulatory mandates, it would be prudent to envision a structure considering the country's cultural context, infrastructure, and resources. Despite facing similar challenges such as limited manpower and resources, several African countries have achieved higher regulatory maturity, as per the WHO global benchmarking tool. This achievement is a result of dedicated autonomous regulatory bodies, transparent quality management systems, ongoing professional training, internal audits, and independent review processes. India, despite holding a higher status for vaccines, has not managed to achieve a higher maturity level for medicines.

The time is ripe for India to envision a framework which prioritises patient safety. An autonomous body governed by board members who are representatives of the community, including doctors, patients, pharmaceutical representatives, and drug controllers, can serve as an ideal structure to implement strong regulation. It should include provisions for mandatory audits, transparency in reports, and a robust drug recall mechanism. The fiscal needs of the regulatory body should be sufficiently met and its functions clearly defined. The regulatory body should strive to maintain an active ongoing association with medical professional organisations, patient support groups, and civil society organisations to uphold public trust, ensure accountability, and foster inclusive, patient-centred regulatory practices. Manufacturers should be bound to stringent regulatory requirements and held accountable through swift legal actions. Periodic inspections and issuance of GMP certificates should be linked to their licence renewal. Immediate implementation of these measures is crucial to prevent avoidable deaths caused by substandard medicines.

Contamination is not the only issue plaguing the quality of medicines. Other hidden issues like prevalence of substandard and falsified medicines and the influence of pharmaceutical lobbying on prescription practices further hinder patient safety. A deeply corrupt and unethical business practice by certain vested interests is thriving within the lax legal and regulatory framework in the country. The call for action is long gone; it is time to act. Unless India restructures its regulatory body with upscaled investment and infrastructure, the pharmacy of the world will lose its credibility. The cost of inaction is being paid through innocent lives; will the country step up?

